# From Networks to Traveling Waves and Back: Persistent Pattern Generation Across Collective Phases in *C. elegans*

**DOI:** 10.64898/2026.06.02.729596

**Published:** 2026-06-05

**Authors:** Assaf Pertzelan, Surabhi Sudevan, Siyu Serena Ding

**Affiliations:** 1 Research Group Genes and Behavior, Max Planck Institute of Animal Behavior, 78464 Konstanz, Germany; 2 Centre for the Advanced Study of Collective Behaviour, 78464 Konstanz, Germany

## Abstract

Thousands of *C. elegans* placed adjacent to a food patch self-organize sequentially into a visible branching network off-food and then a coherent traveling consumption wave — a multi-phase collective phenomenon we term *wurmuration*. Dynamical networks and traveling waves have previously only been described separately in this organism. Using a minimal agent-based model, we show that a single generative program produces both collective phases and their topological continuity. Four components (elongated body geometry, roaming-dwelling switching, preferred local worm density navigation, and food chemotaxis) are necessary and sufficient; what changes between phases is only the food environment they act on. We show that network-like organization persists cryptically (visible only through temporal integration) across the qualitatively distinct wave phase, indicating that the two phases differ in environmental context rather than in underlying behavioral rules. Finally, tuning the preferred density component alone recapitulates N2 vs. DA609 differences in wave dynamics; N2 additionally shows greater per-trial variability in cryptic network topology than DA609, in line with less consistent collective engagement in this lower-sociality strain. Our findings suggest that complex multi-phase collective phenomena can be unified by a shared generative program, shifting the description of spatial organization from the emergent patterns to their underlying components.

## Introduction

The same local interaction rules can produce qualitatively distinct macroscale collective patterns, a principle documented across biological scales and taxa ^1,2^. In *Dictyostelium*, the same cAMP-directed chemotaxis rules generate both dispersed solitary foraging and organized collective aggregation ^3^; in desert locusts, local alignment rules underlie both solitary disordered movement and coherent directed marching^4^; in fish schools, the same interaction rules, operating under shifting parameter regimes, produce polarized schooling, milling, and disordered swarming ^5^. In *C. elegans*, collective patterns emerge from the interplay between population density, food availability, and ambient oxygen levels ^6–10^, producing a repertoire of collective behaviors including stationary dense-cluster aggregation ^9^, motile collective swarming^7,10–12^, dynamical network remodeling^6^, and density- and food-dependent pattern transitions^10^. Ding, Schumacher et al.^7^ showed that density-dependent speed switching, reversals and neighbor-taxis can reproduce both static aggregation and motile swarming from this minimal rule set, establishing that a shared generative program underlies these collective behaviors. While collective swarming has been reported across *C. elegans* population scales^7,10–12^, it has only been modeled in small populations^7^. At large population scales, *C. elegans* collective behavior exhibits a fundamentally distinct phenomenology, encompassing multiple collective patterns within a single behavioral episode. No quantitative or mechanistic account exists to describe the emergence of and the transition between these patterns, nor to unify their relationship with previously described aggregation, swarming, and network remodeling.

Here we formally describe *wurmuration*, a large-scale, multi-phase collective phenomenon in *C. elegans*. When thousands of adult worms are deposited adjacent to a bacterial food patch, they first self-organize into a visible branching network in the off-food reservoir (network phase), then transition to a coherent traveling wave (wave phase), in which the network topology is not obvious from instantaneous observation. Both phases of wurmuration are purely emergent; no physical template connects them. Dynamical networks^6^ and collective swarming^10,11^ have each been documented in *C. elegans*, but their sequential emergence as phases of the same behavioral episode, and the generative basis of their relationship had not been established. By developing an agent-based model, we show that four components — elongated body geometry, roaming-dwelling switching, preferred local worm density (PD) navigation, and food chemotaxis — are necessary and sufficient to produce both wurmuration phases and their topological continuity. A single between-strain parameter, PD, recapitulates the observed differences in wave dynamics between the lab reference strain N2 and the collective mutant DA609.

The wave phase, examined more closely, contains a further finding. Temporal integration of worm positions reveals a network-like occupancy imprint whose topology corresponds to that of the network phase, indicating that network-like spatial organization re-emerges during the wave phase. In several systems, network structures are known to persist cryptically within a collective phase: Lagrangian coherent structures reveal transport networks undetectable in apparently unstructured flows^13,14^; the brain’s functional connectivity network persists through physical synaptic architecture as traveling waves dominate during sleep ^15^; contagion waves in fish schools propagate through hidden interaction networks that directly govern wave dynamics^16^. In each of these cases, networks and waves are linked by physical structure or functional dependence. By contrast, our wurmuration model reproduces network-like imprint during the wave phase simply as a mechanical consequence of the same four underlying components. We demonstrate that both wurmuration phases, and the cryptic continuity of network organization across them, emerge from a single set of components operating in changing environmental contexts, suggesting that the relevant level of description for this multi-phase collective organization lies in the shared generative components rather than in the emergent patterns they produce.

## Results

### Wurmuration exhibits multi-phase spatiotemporal dynamics

**Figure 1.**
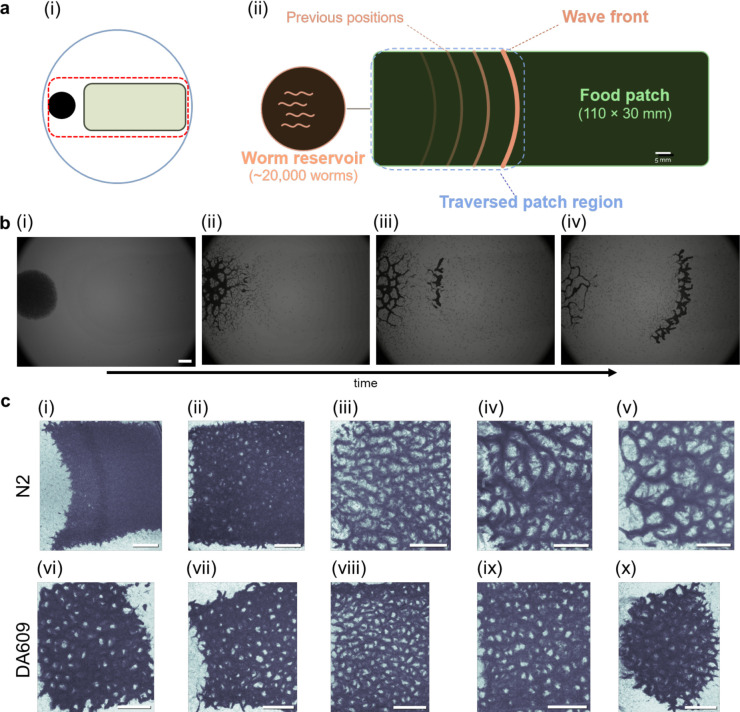
Wurmuration exhibits visible and cryptic network-like spatial organization across its two phases. **a** (i) Schematic of the experimental setup in the 150 mm-diameter behavioral arena. The worm reservoir (black circle) is positioned at the left edge of the bacterial lawn (green rectangle), with the detailed view area indicated by the dashed red rounded rectangle. (ii) Detailed view of the interaction area. Individuals may navigate inside the Petri dish beyond the boundaries of this view. Figures not drawn to scale. **b** Time-lapse sequence showing: (i) initial reservoir, (ii) network phase: visible branching network in the worm reservoir , (iii) network-to-wave transition: network draining and wave emergence, (iv) wave phase: traveling wave propagation across the bacterial lawn. **c** Representative TCIs showing cryptic network-like spatial coverage during wave phase. Dark bundles indicate worm-traversed regions; bright areas indicate less visited regions. (i-v) N2 strain showing variable spatial coverage across trials from dense, continuous coverage to sparse cryptic networks. (vi-x) DA609 strain showing more uniform cryptic network topology and hole size distribution across trials. All scale bars: 5 mm.

To characterize wurmuration, we deposited ~20,000 synchronized Day 1 *C. elegans* adults in a concentrated droplet adjacent to a ~110 × 30 mm rectangular *E. coli* OP50 lawn (Fig. 1a). Time-lapse imaging revealed a stereotyped progression of collective dynamics (Fig. 1b; Supplementary Video S2a, c). Initially, worms formed a network-like structure within the off-food worm reservoir as the liquid absorbed into the hydrogel media (network phase) (Fig. 1b–ii). This network subsequently drained towards the food patch, giving rise to a coherent traveling wave propagating across the bacterial lawn (wave phase) (Fig. 1b–iii,iv). We use the term ‘phase’ throughout in the biological sense to refer to macroscopically distinct collective behaviors, rather than in the thermodynamic sense of an order-parameter transition. Close observation of wave phase dynamics revealed that the wave front did not sweep uniformly across the food patch: worms appeared to preferentially follow along certain routes, channeling around discrete areas as thick bundles rather than passing through them, leaving those areas progressively encircled but unvisited as the front advanced, a hint that spatial patterning persisted during the wave phase (Supplementary Video S1).

To reveal worm positions across the entire wave phase we generated images in which each pixel retains the darkest value recorded at that location across all frames, marking regions of peak worm occupancy. These temporal composite imprints (TCIs; Fig. 1c) displayed a distinctive network-like topology, with dark bundles indicating worm-traversed regions and bright areas representing less visited regions. This cryptic network-like coverage, invisible in instantaneous frames but recoverable through temporal integration, was observed robustly across both the lab reference strain N2 and the collective mutant DA609 (Fig. 1c). The emergent pattern was further observed across a broad range of strain genetics, population compositions, arena sizes and food conditions (Supplementary Fig. S1), demonstrating that network-like spatial organization is a general and consistent feature of wurmuration, both as a visible network in the off-food reservoir and as a cryptic network during on-food wave propagation. N2 TCIs (Fig. 1c, i–v) showed greater per-trial variability in wave phase coverage topology compared to DA609 (Fig. 1c, vi–x).

### Agent-based simulations recapitulate experimental multi-phase dynamics

To investigate the minimal requirements for the observed collective dynamics, we developed an agent-based model. In the model, self-propelled agents representing individual worms are initialized in a dense off-food reservoir adjacent to a rectangular food patch. Each agent senses its local neighborhood, switching between dwelling and roaming states depending on local worm density, and navigates stochastically toward regions of preferred local worm density (PD), with a probability of redirecting toward the food source that increases with food odor strength. Upon reaching the patch, chemotaxis is suppressed and agents consume food, progressively depleting it and shifting the food gradient; accumulation at the patch boundary brings local density to PD, driving dwelling and producing the wave front.

**Figure 2.**
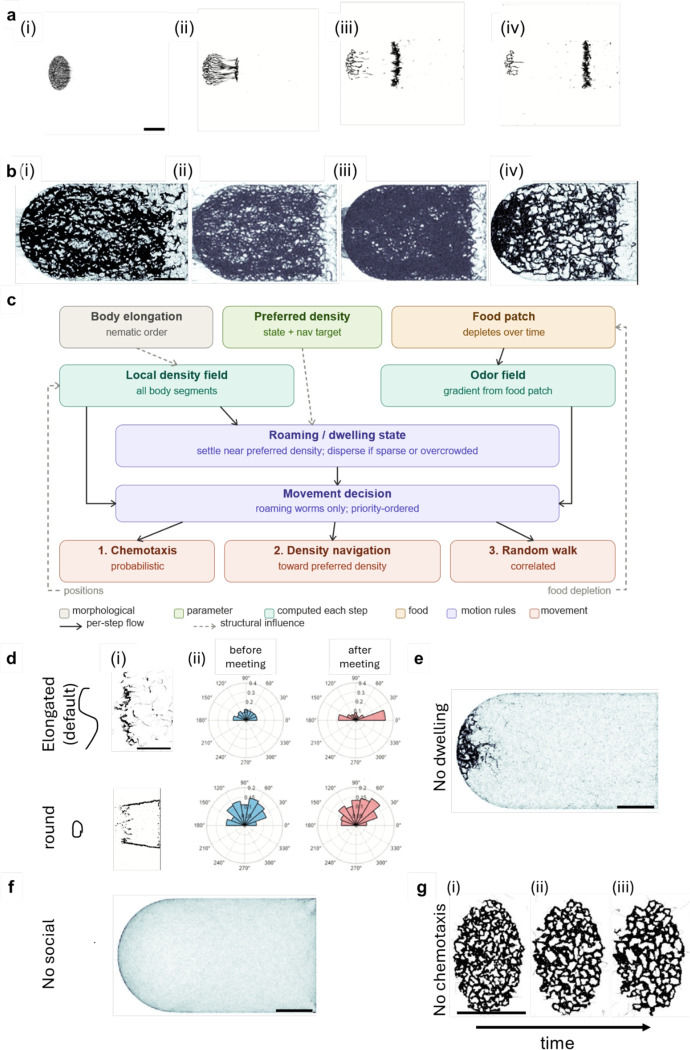
Agent-based simulations recapitulate experimental multi-phase dynamics. **a** Replication of the observed wurmuration dynamics from visible network formation in the worm reservoir to traveling wave initiation and propagation. Compare to Figure 1b.**b** Multiple examples of spatial imprint of simulated agent positions over full simulation, showing persistent occurrence of cryptic network-like coverage pattern across different parametrizations. Compare to experimental TCIs in Figure 1c. **c** Model architecture schematic; see Supplementary Methods, Agent based Simulation for full description. **d–g** Necessity of each model component (Supplementary Video S3; Supplementary Methods, Mechanism tests).**d** (i) TCI comparison of elongated vs. round agents. **d** (ii) Angular distribution of pairwise approach and departure angles, corrected for geometric sampling bias (see Methods, Simulation Analysis; Supplementary Methods, Mechanism tests). No explicit alignment rule is implemented; nematic order emerges solely from body elongation. **e** TCI, roaming-only agents (no dwelling). **f** TCI, no PD navigation. **g** Time-lapse, no food chemotaxis. All scale bars: 5 mm.

The model successfully recapitulated the complete wurmuration sequence from the visible network phase through traveling wave emergence and propagation ( Fig. 2a, compare to Fig. 1b; side-by-side comparison in Supplementary Video S2). Strikingly, the simulations also reproduced the cryptic network-like spatial coverage patterns observed in experimental TCIs (Fig. 2b, compare to Fig. 1c), demonstrating that our model reproduces the observable collective dynamics and patterns; quantitative comparison of structural metrics between experiments and simulations follows in subsequent sections. The dependency structure of all four components is shown in Fig. 2c to illustrate how they combine at each step to produce individual behavior (see also Supplementary Methods, Numerical illustration of component interactions).

By conducting leaving-one-out analyses, we determine that four model components are necessary and sufficient (Fig. 2d–g; Supplementary Video S3; Supplementary Methods, Mechanism tests). *Body elongation* generates nematic order: elongated agents became strongly aligned after pairwise interactions while round agents did not, and without elongation the patch was consumed in a uniform advancing front (Fig. 2d; Supplementary Methods, Mechanism tests). *Roaming-dwelling switch* is essential for sustaining the network structure: the agents generate the structure while roaming and the switch to dwelling keeps it stable. Roaming-only agents produced transient fronts that subsequently fragmented ( Fig. 2e). *Preferred local worm density navigation*, where agents settle near moderate local crowding and disperse from both local density extremes, is required for the network bundle formation; removing it yields uniform coverage (Fig. 2f). *Food chemotaxis* converts density-driven dynamics into a directed wave; without it, agents form continuously remodelling networks but no coherent front (Fig. 2g), resembling the dynamic network states reported by Sugi et al. ^6^. Together, these four model components produce the cryptic network-like TCI signature through their interaction with local food depletion.

### Varying preferred density captures strain differences in wave dynamics but not cryptic network topology

Having established that the general wurmuration behavioral sequence emerges from a minimal set of components, we next asked what parameter variation accounts for the observed differences between strains. DA609 [*npr-1(ad609)* null] is a collective mutant that preferentially aggregates at higher local worm densities, partly mediated by a preference for the local low-oxygen microenvironments created by conspecific respiration and limited oxygen diffusion in dense groups ^8,9,17^. In our model, this enhanced density-seeking tendency is represented by a higher PD, the local worm density at which agents dwell stably. We therefore set PD = 6 for N2 and PD = 12 for DA609 with all other parameters held constant, reflecting the relative difference in density-seeking tendency between strains to test whether this single parameter variation explains the strain differences (Supplementary Video S2, panels a–d). Our simulations seek to reproduce structural patterns and relative trends between the strains rather than absolute experimental values (see Supplementary Note S1).

**Figure 3.**
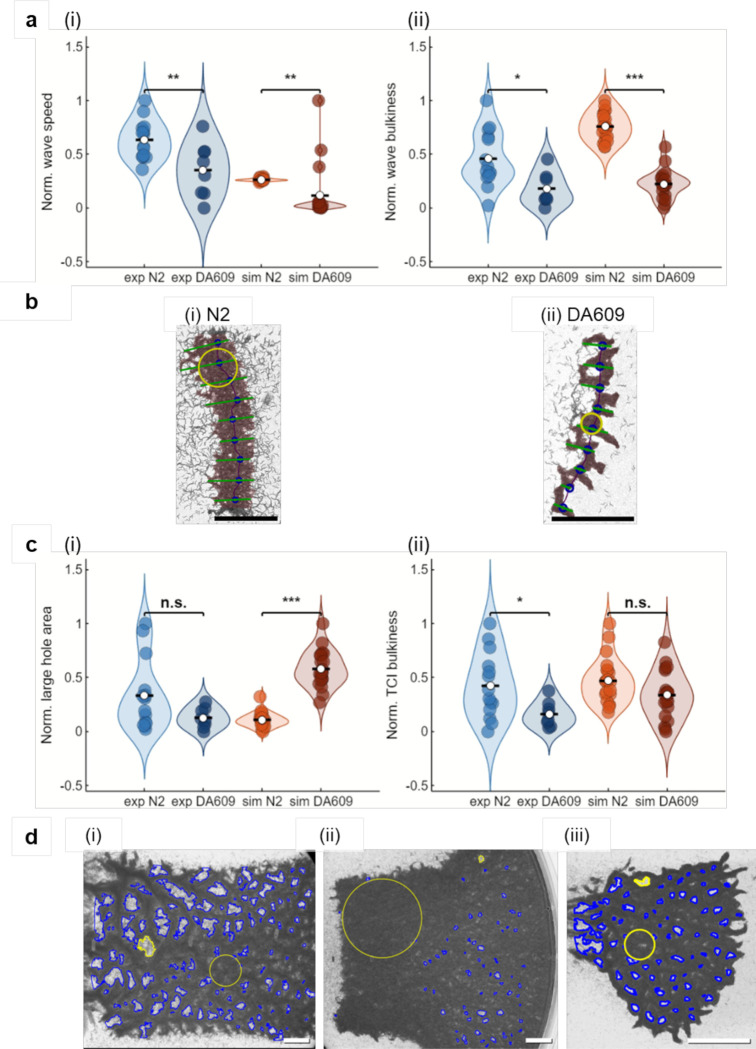
Varying PD parameter recapitulates strain-specific wave dynamics but not cryptic network patterns. **a** Wave properties in experiments (blue) and simulations (orange). Simulated N2 (PD=6) and DA609 (PD=12) differ only in PD. Values are independently min–max normalized to [0–1] over the combined N2 and DA609 range within each dataset pair for visual comparison only; all statistical tests were performed on raw values; (i)*wave speed,* (ii) *wave bulkiness.* Statistical tests use raw values (Supplementary Table S2). *: p<0.05; **: p<0.01; ***: p<0.001. Experimental sample sizes are: N2 *n* = 14, DA609 *n* = 8; simulation sample sizes are: N2 *n* = 20, DA609 *n* = 20. **b** Representative wave morphologies showing N2 wave and DA609 wave. Yellow circles indicate *wave bulkiness*; green lines show sampled wave cross-sections (*wave width*; see Supplementary Methods, Wave properties extraction). **c** Cryptic network properties in experiments (blue) and simulations (orange). Values are independently min–max normalized to [0–1] over the combined N2 and DA609 range within each dataset pair for visual comparison only; all statistical tests were performed on raw values; (i)*large hole area*, (ii) *TCI bulkiness*, Statistical tests use raw values (Supplementary Table S2). *: p<0.05; ***: p<0.001; n.s.: not significant. Experimental sample sizes are: N2*n* = 14, DA609 *n* = 8; simulation sample sizes are: N2 *n* = 20, DA609 *n* = 20. **d** Representative experimental TCIs: (i) N2 with high *large hole area* and low *TCI bulkiness*, (ii) N2 with low *large hole area* and high *TCI bulkiness*, (iii) DA609 with more uniform coverage across trials. Yellow regions are the 5th largest hole and the bulkiness radius. All scale bars: 5 mm.

This single PD difference recapitulated the observed strain differences in wave dynamics (Fig. 3a; Supplementary Table S2). In both experimental data and simulation sets, N2 *wave speed* is higher than DA609 (experiments *p* = 0.007, simulations *p* = 0.006; See Supplementary Table S2 for all statistics), and N2 waves are broader than DA609 as measured by *wave bulkiness*, the maximal inscribed radius of the wave cross-section (Fig. 3b yellow circles; experiments *p* = 0.022, simulations *p* < 0.001). The agreement in *wave speed* and *wave bulkiness* across both experimental data and simulation sets supports the interpretation that varying PD alone is sufficient to capture strain-specific differences in wave dynamics (See Supplementary Note S1 for the quantitative scale difference between experiments and simulations).

However, varying the PD parameter alone was insufficient to reproduce the observed differences in cryptic network topology between strains (Fig. 3c; Supplementary Table S2). While experimental TCIs showed higher *TCI bulkiness* in N2 than in DA609 (*p* = 0.030) and no difference between strains in *large hole area* (*p* = 0.164) (Supplementary Methods, Network Property Extraction), simulations produced no difference in *TCI bulkiness* (*p* = 0.066) but higher *large hole area* in DA609 than in N2 (*p* < 0.001). This disagreement between experimental and simulation results for network topology metrics could be due to pronounced per-trial variability observed in N2 but not DA609 experimental data, which is not currently captured by the model simulations. Figure 3d shows representative experimental TCIs illustrating different combinations of the two network metrics: N2 with high *large hole area* and low *TCI bulkiness* (Fig. 3d–i), N2 with low *large hole area* and high *TCI bulkiness* (Fig. 3d–ii), and DA609 with more uniform coverage across trials (Fig. 3d–iii). We further characterized and modeled N2 per-trial variability in the following section.

### N2 shows pronounced per-trial variability in cryptic network topology

**Figure 4.**
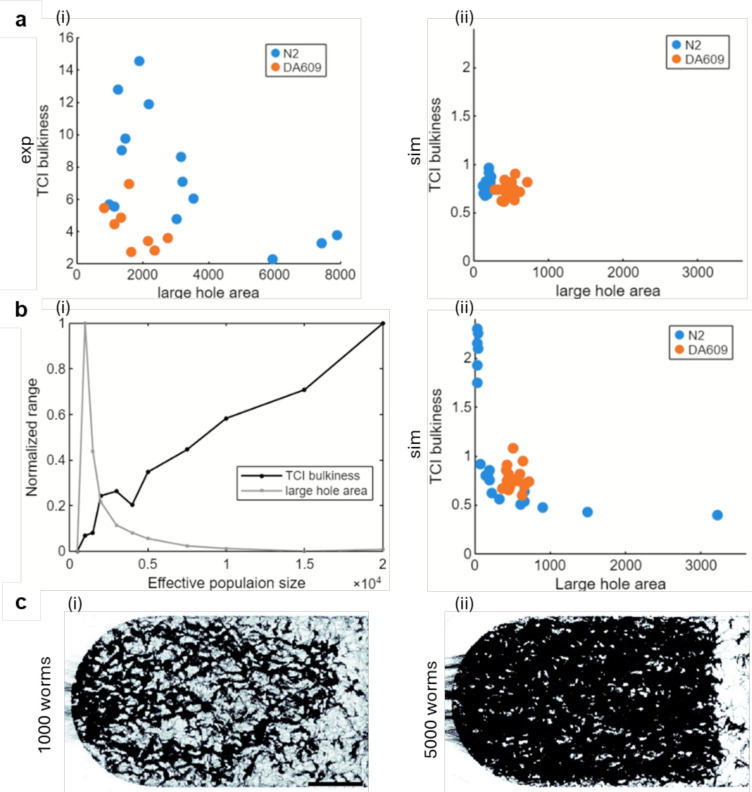
Pronounced N2 per-trial variability in cryptic network topology distributes along a topological coverage trade off. **a** Scatter of *large hole area* vs. *TCI bulkiness* for (i) experiments and (ii) simulations at fixed PDs. N2 standard deviation is ~3.5-fold that of DA609 for *large hole area* (p = 0.032) and ~2.6-fold for *TCI bulkiness* (p = 0.023); permutation p-values are in Supplementary Table S2. **b** Effective population size variation as an illustrative source of per-trial variability within N2. (i) Simulations across a range of effective population sizes (all other parameters at default) show opposing trends in *TCI bulkiness* (black) and *large hole area* (gray). (ii) Introducing effective population size variation into N2 simulations (low PD) reproduces the experimental scatter pattern (compare to a-i); DA609 simulations (PD = 12) use the same parameterization as those shown in Fig. 4a.-ii**c** Representative N2 simulation TCIs from (i) small (1000 agents) and (ii) large (5000 agents) populations. Sample sizes: n = 11 per effective population size value. Scale bar: 5 mm.

N2 experiments showed substantially greater per-trial variability in cryptic network topology than DA609 experiments. Standard deviations for network metrics are 3.5-fold higher in N2 ( p = 0.032) than DA609 for *large hole area,* and 2.6-fold higher in N2 than DA609 for *TCI bulkiness* (p = 0.023) (permutation tests on SD ratio; Supplementary Table S2; see Methods). The two metrics are negatively correlated within N2 trials (Spearman ρ ≈ −0.53), reflecting a geometric trade off where denser network bundle coverage necessarily reduces the area available for unvisited holes while sparse network coverage leaves more room for them. Approximately 60% of N2 trials (8 of 14) fall outside the DA609 range for at least one network metric, distributing either toward high *large hole area* or high *TCI bulkiness* but not both. DA609 trials occupy a substantially tighter region in this two-metric space, with no analogous per-trial scatter (Fig. 4a–i). By contrast, simulations failed to replicate these phenotypic differences, yielding comparable variability between strains ( Fig. 4a–ii).

To show that a single source of biological variability could account for this scatter in N2, we used effective population size as an illustrative example. Effective population size captures the possibility that different proportions of the total N2 population participate in collective movement across trials, an intuitive source of variation for a less social strain. Varying effective population size in our N2 simulations produced opposing trends in *TCI bulkiness* and *large hole area* (Fig. 4b–i), as well as the observed scatter in N2 experiments (Fig. 4b–ii; representative TCIs in Fig. 4c).

Together, these results show that N2’s per-trial variability in cryptic network topology is organized along a topological coverage trade off that is consistent with variation in the effective population size. These results suggest that N2 populations produce more variable emergent spatial organization, potentially due to the strain’s less consistent sociality tendency to engage in collective movement. DA609, by contrast, produces a more stereotyped emergent pattern across trials, consistent with stronger sociality constraining collective outcomes.

## Discussion

We describe wurmuration, a large-scale multi-phase collective phenomenon in *C. elegans* in which the same population sequentially self-organizes into a visible branching network and then a coherent traveling consumption wave within a single behavioral episode. The sequential emergence of the network and wave phases, each previously described only in isolation in this organism ^6,10,11^, constitutes our primary phenomenological finding. Temporal integration of the wave phase renders cryptic collective patterns accessible, recovering a network-like spatial organization hidden from instantaneous observations alone. These findings together call for a mechanistic account that can encompass both collective phases and their spatial relationship within a single framework.

Our mechanistic framework establishes that four interacting components — elongated body geometry, roaming-dwelling switching, PD navigation, and food chemotaxis — are necessary and sufficient to generate both wurmuration phases. What changes between the phases is not these four components but the environment they act on: food-absent in the network phase, food-present and depleting in the wave phase. The model reproduces the complete behavioral sequence, captures directional strain differences across both primary wave-phase metrics, and recapitulates the characteristic per-trial variability structure of N2 network topology, providing a mechanistically grounded account of how two visually distinct collective phases arise from the same generative components. The quantitative gap between model and experiment, the dynamics of the network-to-wave transition itself, and the sources of N2 per-trial variability are addressed in Supplementary Note S1 and Supplementary Note S2.

The strain comparison positions PD as a genetically tunable axis of the four-component wurmuration framework. Varying it as the single between-strain parameter recapitulates directional differences across both primary wave metrics, with higher PD producing slower, more compact waves in DA609 than in N2. Beyond wave dynamics, N2 experiments show substantially greater per-trial variability in cryptic network topology than DA609, with an inverse relationship between *TCI bulkiness* and *large hole area* metrics reflecting geometric trade offs in coverage characteristics. DA609 experiments produce a far more stereotyped network metric distribution across trials than N2, occupying a compact region in this two-metric phenotypic space, consistent with stronger sociality constraining collective outcomes. We hypothesized that the higher per-trial variability of N2 could be due to less constrained collective engagement from trial to trial in this less socially inclined strain, and supported this interpretation with an additional parameter in the N2 simulations to recapitulate the variable cryptic network characteristics seen across N2 experiments. While varying effective population size in our N2 simulations succeeded in explaining the observed per-trial variation, this new parameter provides one potential illustration for the role of sociality in constraining collective behavioral stereotypy. The actual biological source and mechanisms driving the per-trial variation in low sociality N2 remains an open question for future investigation.

The density-centric logic underlying wurmuration connects with the framework established by Ding, Schumacher et al.^7^ across several orders of magnitude in collective scale. Where that work established that density-dependent speed state switching and neighbor-taxis reproduce aggregation and swarming in small populations, the same density-centric logic here encompasses large-population network formation and directed wave propagation within a single behavioral episode. We further extended this previous work in two specific directions. First, the neighbor-taxis rule required for cohesion in that model is here subsumed into PD navigation alone, suggesting that movement toward preferred-density regions may be sufficient for both state-switching and directional cohesion without a separate attraction term. Second, while Ding, Schumacher et al. showed that local food depletion drives aggregate motility in the swarming phase, food chemotaxis in our model acts as an explicit active component converting density-driven network dynamics into a directed wave. Here, worms navigate towards food ahead of the wave rather than disbanding passively from depleted regions; the wave’s progressive depletion of local food thus sustains food gradient-directed collective advance without any change in the four components themselves.

Comparing the wurmuration model to other *C. elegans* collective behavior frameworks clarifies the specific mechanistic contribution of each component to network topology and wave formation. Chen and Ferrell ^3^ showed that density-thresholded aggregation can arise from purely passive condensation: worms performing random walks slow upon colony contact, without any active taxis toward conspecifics producing density-dependent clustering. The spatial outcome is round, liquid-like aggregates, not branching networks, establishing that network topology requires active spatial patterning that passive velocity reduction alone cannot provide. Sugi et al.^6^ identified a candidate: density-dependent social aggregation combined with nematic alignment generates branching dynamic networks in the absence of food. Critically, nematic ordering was an empirically observed property in their system and encoded as an explicit alignment rule in the model. In our wurmuration model, nematic organization is not imposed fixed input but an emergent mechanistic output: multi-segment elongated worms with directional persistence generate lane-like alignment under PD constraints without any explicit alignment interaction, as demonstrated by comparison with isotropic round agents (Fig. 2c). The four-component wurmuration framework thus mechanistically identifies body geometry as the source of nematic order, a property that Sugi et al.’s framework presupposes and Chen and Ferrell’s does not require. Food chemotaxis, absent from both prior frameworks, then completes the account by converting continuous network remodeling into directed wave propagation within the same behavioral episode in wurmuration.

Our four-component framework accounts directly for the visible and cryptic networks during wurmuration. In the off-food reservoir, PD navigation and elongation-induced nematic ordering together produce the visible network structure; in the wave phase, the same components continue to operate, now under the additional constraint of a food gradient that directs collective movement forward. The cryptic network imprint during the wave phase is therefore not a memory or preservation of the network phase, but a fresh production of the same spatial process in a changed environmental context. Removing food chemotaxis from the model recovers continuous network remodeling without directed wave propagation — closely resembling the dynamic network states described by Sugi et al. ^6^— confirming that food chemotaxis is specifically what converts density-driven network dynamics into the directed traveling wave. In this sense, the wave-network relationship observed in our system is closer in character to mycorrhizal fungi systems where the advancing hyphal front and the network it constructs are related aspects of the same ongoing process^18^, than to systems where cryptic networks are either physically or functionally preserved during waves^15,16^. In *C. elegans* regimes where very high population density or neuromodulatory tuning of social state dominate, no network structure forms in either collective phase ^10–12^, marking the outer limits of the parameter space in which the four components produce networked spatial organization. Within those limits, the breadth of conditions under which the network-like TCI signature appears during wurmuration supports the broad emergence of this spatial pattern across genetic backgrounds, population compositions, arena sizes, and food conditions (Supplementary Fig. S1) when the four components operate together on a food substrate.

In wurmuration, the four mechanistic components cast the same topological shadow regardless of the environmental context; what changes between the network phase and the wave phase is only the visibility of that shadow. This finding has implications beyond *C. elegans*: where distinct collective phases are governed by a single set of generative components, the spatial organization of one phase may be cryptically present in another — invisible to direct observation but recoverable through the right measurement perspective. Collective spatial organization may therefore sometimes be more informatively described at the level of the generative components than at the level of the patterns they produce.

## Methods

### Behavior experiment and video acquisition

#### Experimental Procedures.

*C. elegans* strains N2 (lab reference strain) and DA609 (*npr-1(ad609)*) were cultured at 20°C on NGM plates seeded with *E. coli* OP50. Synchronized Day 1 adult populations were obtained by alkaline-hypochlorite treatment of gravid adults to isolate eggs, and letting eggs hatch overnight in M9 at 20°C on a rotator. L1 diapause larvae were refed on OP50 plates for 65 hours to reach adulthood. Imaging plates were prepared by pouring 200 mL of no-peptone NGM agar into 150 mm-diameter glass Petri dishes (Rotilabo KKA1.1, Carl Roth), and drying overnight on the bench. Rectangular food lawns (~110 × 30 mm) were created by spreading 3 mL of OP50 (OD_600_ ≈ 2.0) with a glass spreader. Seeded imaging plates were allowed to dry for 2–3 h at room temperature and incubated overnight at 37°C, before returning to room temperature prior to the experiment. For each experiment, ~20,000 synchronized Day 1 adults were harvested from culture plates and washed in M9 to remove bacteria. After the final wash, worms were concentrated to a ~200 μL pellet by centrifugation (Eppendorf Centrifuge 5702) at 2,000 rpm for 2 min, and deposited at the left edge of the food lawn using a glass pipette. After letting the worm pellet dry for 10–15 min, plates were transferred to an Axio Zoom V.16 microscope (Zeiss) for behavioral imaging.

#### Video Acquisition.

Bright-field imaging was performed at 20°C using an Axio Zoom V.16 microscope (Zeiss) equipped with an Axiocam 712 colour camera (Zeiss) and an HXP 200C light source (Zeiss). Imaging with a PlanApo Z 0.5x objective at 3.5x magnification gives a field of view of 68×51 mm. Time-lapse videos were acquired at 0.1 fps with 0.5 ms exposure at 70% light intensity using ZEN 3.4 Pro software (Zeiss). Fourteen independent experiments were performed for N2 and eight for DA609 across multiple experimental days. Trial durations ranged from 90 to 335 min and were statistically indistinguishable between strains (N2: 161 ± 56 min; DA609: 193 ± 76 min; p = 0.28, two-sample t-test); all primary metrics were validated to be independent of trial duration (Supplementary Methods, Network Property Extraction; Supplementary Fig. S2).

### Video Processing and Analysis

#### Wave Property Extraction.

Wave temporal boundaries were identified from the mode first-visit frame index at the lateral extremes of the food patch, refined by manual annotation where needed. Wave masks were extracted at 10-minute intervals by morphological processing of binarized frames; centerlines were computed as row-wise averages of mask pixels, smoothed by moving average. *Wave speed* was measured from the lateral displacement of wave boundaries over the annotated time span; *wave bulkiness* is the maximal inscribed radius of the wave mask. Full details are in Supplementary Methods, Wave Property Extraction.

#### Network Property Extraction.

The TCI for each trial is generated by stacking all video frames through temporal minimum projection: each pixel retains the darkest value it ever reached across the experiment, corresponding to its moment of peak worm occupancy. To suppress transient single-worm events before projection, frames are temporally median-filtered in a sliding window (6 subsamples per 10-minute window) prior to taking the minimum (parameter choice validated in Supplementary Fig. S3; see Supplementary Methods, TCI parameter validation). Within the manually annotated food-patch boundary, bright (worm-free) regions in the TCI were segmented as holes by Otsu thresholding; components smaller than a minimum area threshold or touching >15% of the patch border were excluded. Network *large hole area* (5th-rank hole by area; rank stability validated in Supplementary Fig. S4) and *TCI bulkiness* (maximal inscribed radius) of the worm-occupied mask were the primary topology metrics. Full pipeline details are in Supplementary Methods, Network Property Extraction.

### Agent-Based Simulation

#### Model construction.

A discrete-time agent-based model was implemented on a 30 × 30 mm grid (20 px/mm). The initial arena features a vertically centered, rectangular food patch with rounded corners on the left side, mimicking the experimental setup. Each agent is a chain of 20 segments (1 mm body length, 50 μm width per segment), capturing the 1:20 aspect ratio ^19^; all segments contribute to local density calculations, so elongated agents imprint a directional footprint on the density field. Forward movement adds a new segment at the head and removes the oldest one at the tail. Agents switch stochastically between dwelling and roaming with probabilities that depend on local density relative to PD; roaming steps follow a priority-ordered hierarchy of odor guidance, density-gradient navigation, and correlated random walk, with angular noise drawn uniformly from [−45°, +45°] added in all cases. How these components interact dynamically is described in Model behavior below. Food depletes progressively at worm heads; when multiple agents occupy the same location, each individual consumes less than it would alone. To represent N2 and DA609 strains we set PD = 6 and PD = 12 segments/pixel respectively, with all other parameters constant (Supplementary Table S1). Necessity of each component was tested by matched simulations removing body elongation, switch to dwelling state, PD navigation, or food chemotaxis in turn. Full model description is in Supplementary Methods, Agent-Based Simulation.

#### Model behavior.

Each time step, a worm is either dwelling or roaming, and may stochastically switch between the two (transition probabilities in Supplementary Methods, Agent-Based Simulation). The following describes how elongated body, roaming-dwelling switch, PD navigation and food chemotaxis interact dynamically across the arena. The switching probability depends on local crowding: dwelling worms in sparse regions are likely to start roaming, roaming worms near their preferred crowd density are likely to stop and dwell, and roaming worms in overly dense regions are unlikely to stop and are pushed back into motion by overcrowding. This single mechanism spontaneously drives the population toward PD without any explicit attraction rule. If a worm is roaming, it then asks, in order: do I smell food and am I not already on it? With a probability that rises with odor strength and falls with local food availability, it roams up the gradient. Worms that do not enter this odor-guided mode look at the eight pixels around their head: if a density gradient exists, they step toward the direction that is closest to PD. If no gradient is found because the neighborhood is uniform or empty, they continue in their current direction with a nudge of noise. Two things change the landscape between steps: the worms themselves, whose collective positions continuously reshape the local worm density field; and food consumption, which carves depletion into the patch and thereby shifts the odor gradient that guides chemotaxis. The body geometry is what makes all of this produce spatial order: elongated worms imprint a directional footprint on the density field, which is what gives rise to nematic alignment and ultimately, network structure.

#### Simulation Analysis.

Three types of simulation sets were generated: baseline comparison sets (N2 and DA609, 20 replicates each), mechanism test sets (each component removed in turn), and an effective population size gradient set (used to model N2 per-trial variability; Fig. 4b). All simulated videos were processed through TCI and metric extraction pipelines identical to those used for experimental data. Nematic alignment was quantified from cross section-corrected angular distributions before and after pairwise interactions (Supplementary Methods, Mechanism tests). For comparisons to experimental data in Fig. 3a,c, metric values within each experimental dataset or simulation set were independently min–max normalized to [0–1] over the combined N2 and DA609 range; statistical tests were performed on raw values. Full statistical analysis details are in Supplementary Table S2.

## Supplementary Material

Supplement 1

Supplement 2

Supplement 3

Supplement 4

## Data Availability

Datasets used in this study will be available at Zenodo upon publication at https://zenodo.org/uploads/20506817, including raw experimental videos, processed datasets (TCIs and per-experiment metric tables), and source data underlying all main and supplementary figures.

## Code Availability

All custom MATLAB codes used in this study are available at https://github.com/SerenaDingLab/Wurmuration_2026, including the agent-based simulation, TCI generation pipeline, wave and network metric extraction, and figure generation scripts.
