# Supplementary material for: From Networks to Traveling Waves and Back: Persistent Pattern Generation Across Collective Phases in C. elegans
Source: bioRxiv. 2026 Jun 5:2026.06.02.729596. Preprint. [Version 1] doi: 10.64898/2026.06.02.729596 (PMC13252393; doi:10.64898/2026.06.02.729596)
Supplement: Supplement 1 [file media-1.pdf]

Supplementary Information

From Networks to Traveling Waves and Back: Persistent Pattern Generation Across Collective Phases in *C. elegans*

Assaf Pertzelan^1, Surabhi Sudevan^1, Siyu Serena Ding1,2*

1 Research Group Genes and Behavior, Max Planck Institute of Animal Behavior, 78464 Konstanz, Germany
2 Centre for the Advanced Study of Collective Behaviour, 78464 Konstanz, Germany
^ equal contributions
* corresponding author. Correspondences to: serena.ding@ab.mpg.de

Supplementary Figures **2**
Supplementary Figure S1. Network-like TCI topology is a robust emergent property of wurmuration across diverse experimental conditions 2
Supplementary Figure S2. Independence of phenotypic metrics from trial duration 3
Supplementary Figure S3. TCI pipeline sensitivity analysis 4
Supplementary Figure S4. Hole-rank metric stability 5
Supplementary Videos **6**
Supplementary Video S1. Wave-phase dynamics with manually placed reference markers 6
Supplementary Video S2. Comparison of experimental and simulated wurmuration dynamics 6
Supplementary Video S3. Control simulations demonstrating the necessity of each model component 6
Supplementary Tables **7**
Supplementary Table S1. Core model parameters 7
Supplementary Table S2. Statistics 8
Supplementary Methods **10**
1. Wave Property Extraction 10
2. Network Property Extraction 10
2.1. TCI parameter validation 11
2.2. Hole-rank metric stability 12
2.3. Independence of phenotypic metrics from trial duration 12
3. Agent-Based Simulation 12
4. Numerical illustration of component interactions 14
5. Mechanism tests 15
Supplementary Notes **16**
Supplementary Note S1. Biological realism of model parameters and acknowledged quantitative limitations 16
Supplementary Note S2. Open questions and directions for future investigation 17

Supplementary Figures

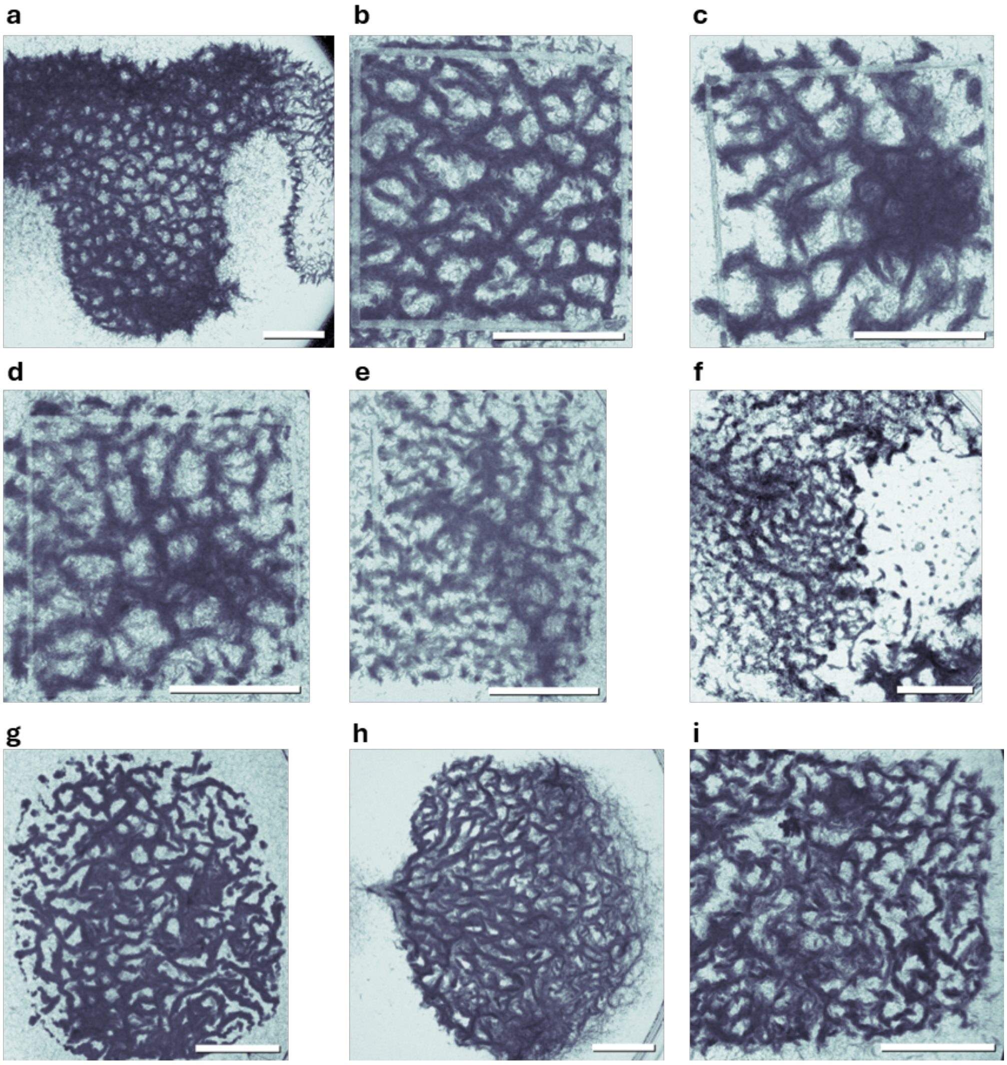

Supplementary Figure S1. Network-like TCI topology is a robust emergent property of wurmuration across diverse experimental conditions. All panels show temporal composite images (TCIs) generated by the same minimum-projection pipeline used for the main dataset (see Methods, Network Property Extraction). Dark regions indicate worm-occupied areas; bright regions indicate less-visited areas. Each panel comes from an independent experiment outside the N2/DA609 main dataset, demonstrating that cryptic network-like coverage is not specific to particular strains or experimental configurations. **a** N2, 20000 Day 1 adults, 150 mm diameter plate, snake-shaped OP50 lawn. **b** DR47 [*daf-11(m47)V*], 10000 Day 1 adults, 50 mm diameter plate, ~20×20 mm square OP50 lawn. **c** JU1249 (wild isolate), 10000 Day 1 adults, 50 mm diameter plate, ~20×20 mm square OP50 lawn. **d** N2, mixed-stage population, 50 mm diameter plate, ~20×20 mm square OP50 lawn. **e** N2, dauer population, 50 mm diameter plate, ~20×20 mm square OP50 lawn. **f** OMG2 [*mIs12[myo-2p::GFP+pes-10p::GFP+F22B7.9p::GFP]II;npr-1(ad609)X*], 4000 Day 1 adults, 50 mm diameter plate, uniform full-plate OP50 lawn. **g** DA609 [*npr-1(ad609)X*], 10000 Day 1 adults, 90 mm diameter plate, ~35x45 mm rectangular lawn of freeze-dried OP50 (LabTIE). **h** OMG2, 12000 Day 1 adults, 90 mm diameter plate, ~45x50 mm rectangular lawn of freeze-dried OP50 (LabTIE). **i** DA609, 10000 Day 1 adults, 50 mm diameter plate, ~25x15 mm rectangular OP50 lawn. All scale bars: 5 mm.

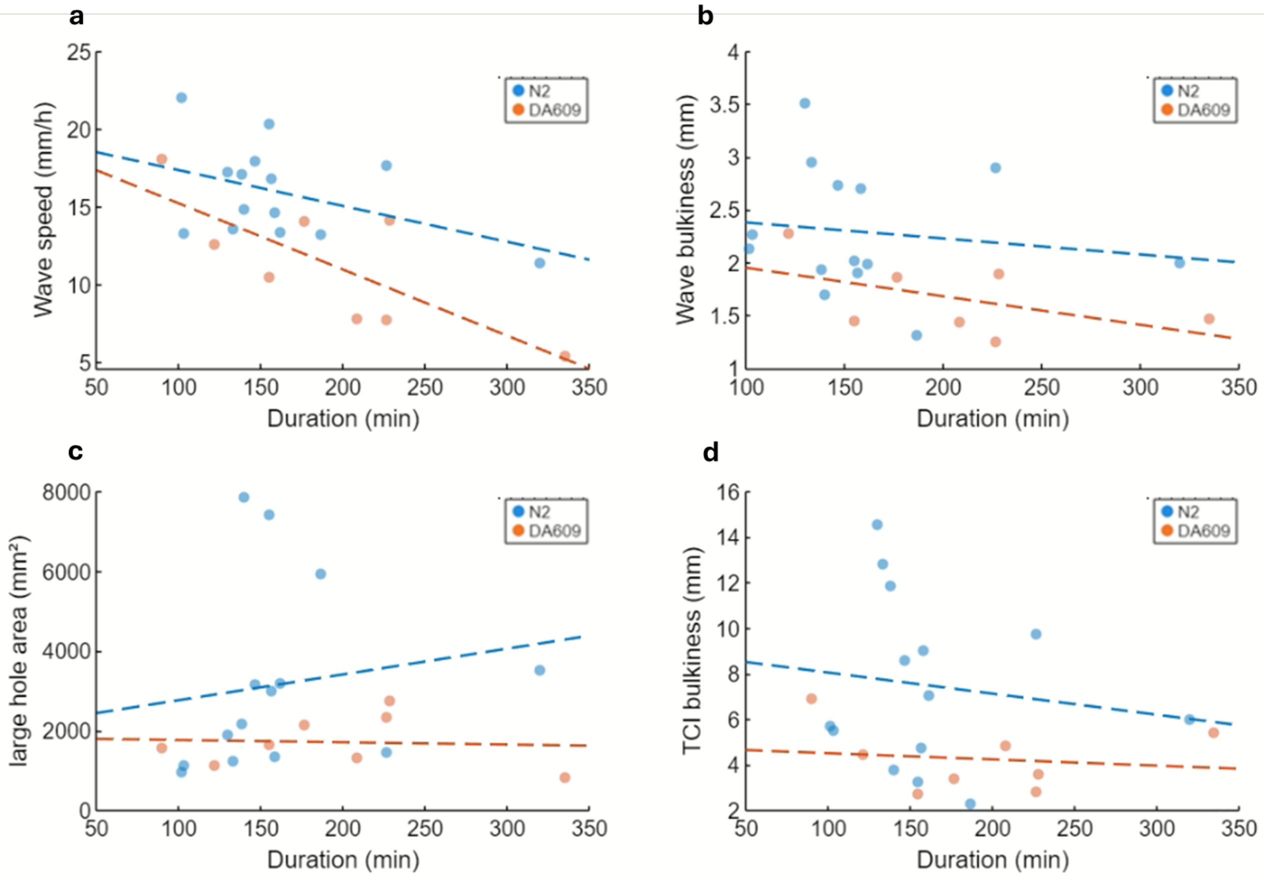

Supplementary Figure S2. Independence of phenotypic metrics from trial duration. Pearson correlation between trial duration and each primary metric, computed separately for N2 (blue, n = 14) and DA609 (orange, n = 8). Dashed lines indicate linear regression fits; R^2 and p-values are reported per strain. **a** *Wave speed* is independent of trial duration in N2 ($R^2 = 0.18$, $p = 0.13$) but shows a significant trend in DA609 ($R^2 = 0.59$, $p = 0.026$). **b** *Wave bulkiness* shows no significant correlation with trial duration in either strain (N2: $R^2 = 0.02$, $p = 0.63$; DA609: $R^2 = 0.26$, $p = 0.24$). **c** *Large hole area* shows no significant correlation with trial duration in either strain (N2: $R^2 = 0.02$, $p = 0.60$; DA609: $R^2 = 0.004$, $p = 0.88$). **d** *TCI bulkiness* shows no significant correlation with trial duration in either strain (N2: $R^2 = 0.02$, $p = 0.64$; DA609: $R^2 = 0.02$, $p = 0.73$). Trial durations are statistically indistinguishable between strains (N2: 161 ± 56 min; DA609: 193 ± 76 min; $p = 0.28$, two-sample *t*-test), confirming that the DA609 *wave speed* trend does not confound the between-strain comparisons reported in the main text.

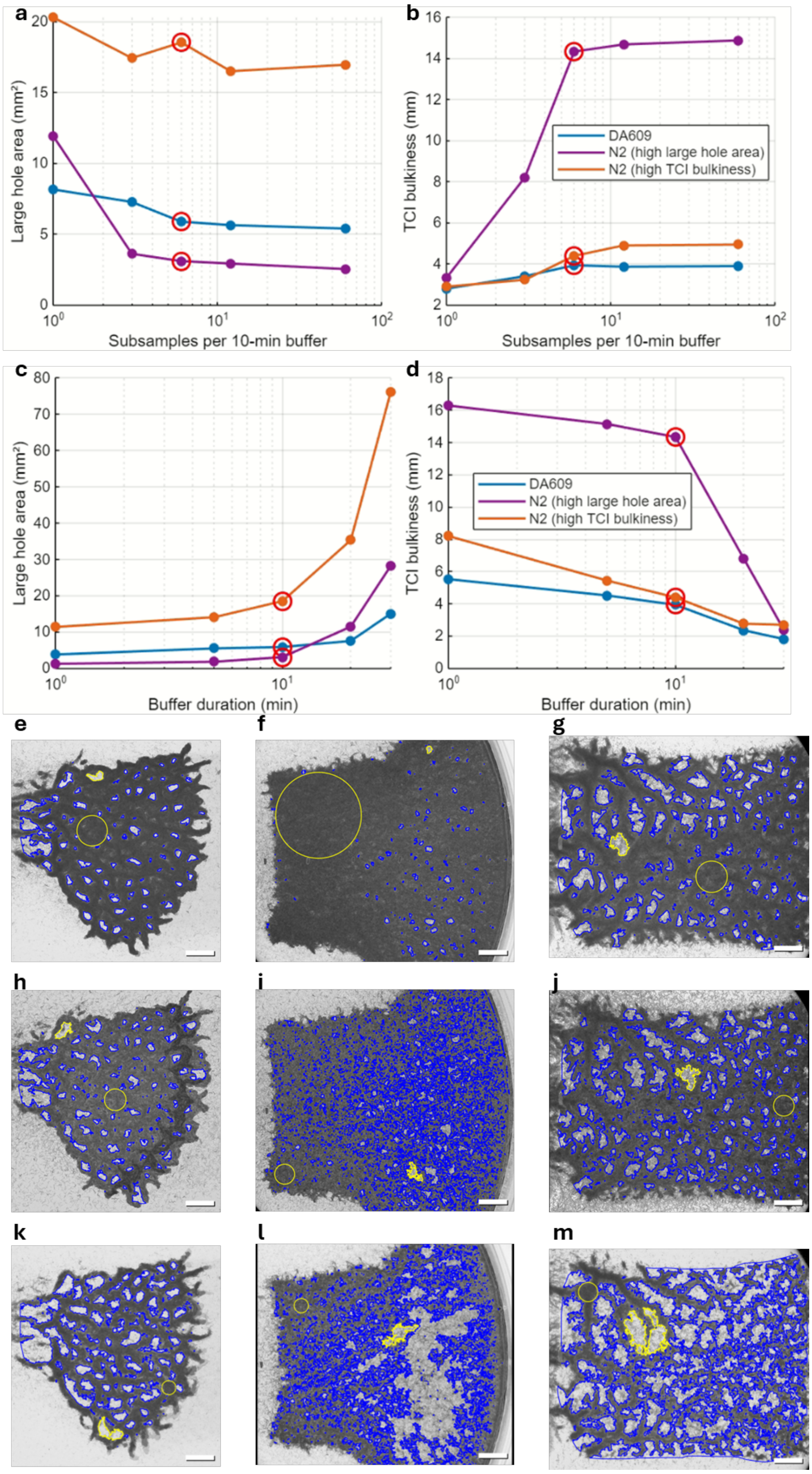

Supplementary Figure S3. **TCI pipeline sensitivity analysis**. **(a-d)** Network metrics (*large hole area*, *TCI bulkiness*) for three representative experiments: DA609 (blue), N2 with high *TCI bulkiness* (purple), N2 with high *large hole area* (orange). Red circles mark the default parameter value. **a–b** Subsamples per 10-minute buffer (buffer duration fixed). **c–d** Buffer duration (6 subsamples fixed). **e–g** Default parameters; 5th-largest hole and *TCI bulkiness* are marked in yellow. **(h–j)** Single-subsample failure mode. **(k–m)** Long-buffer failure mode. See Supplementary Methods 2.1, TCI parameter validation. All scale bars: 5 mm.

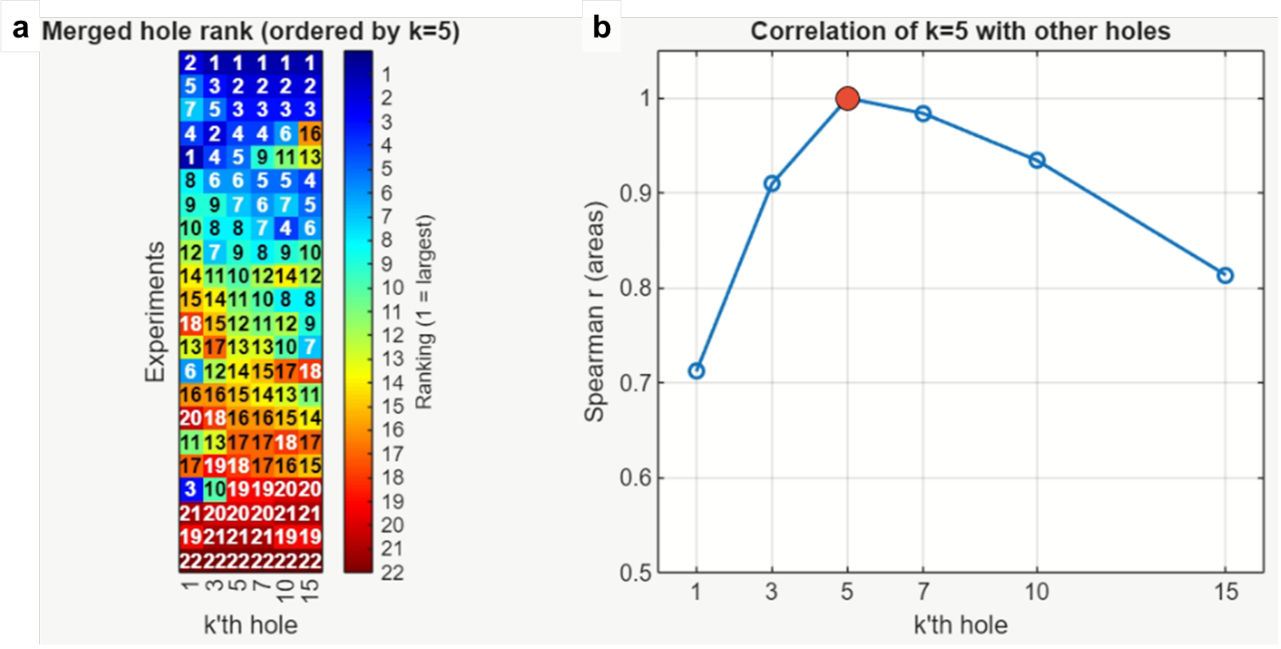

Supplementary Figure S4. Hole-rank metric stability. a Rank matrix for all experiments (N2 and DA609 combined) across hole ranks k = 1, 3, 5, 7, 10, 15, ordered by the k = 5 column. Colour indicates rank within each column (rank 1 = largest area). **b** Spearman correlation between the k = 5 ordering and each k. See Supplementary Methods, Network Property Extraction, Hole-rank metric stability, for interpretation.

Supplementary Videos

Supplementary Video S1. Wave-phase dynamics with manually placed reference markers. Markers were placed on apparent gaps in the advancing wave front prior to playback; their persistence throughout wave progression confirms that these regions remain unvisited. This observation motivated the temporal integration analysis (TCI) in Figure 1c.

Supplementary Video S2. Comparison of experimental and simulated wurmuration dynamics. Panels: **a** N2 experimental, **b** N2 simulated (PD = 6), **c** DA609 experimental, **d** DA609 simulated (PD = 12). Spatial and temporal scales are adjusted across panels to align the onset of the wave phase and standardize panel dimensions. Playback speeds are accelerated by factors of 250, 492, 467, and 600, respectively ; simulation panels are rendered at equivalent density scale (inverted, dark = high worm density). Scale bars and strain/condition labels are indicated on each panel.

Supplementary Video S3. Control simulations demonstrating the necessity of each model component (corresponding to Figure 2d–g). All panels use identical parameters (Supplementary Table S1, 1500 agents) except for the single component removed in each case. Temporal scales are matched across panels. **a** No body elongation. **b** No food chemotaxis (left: simulated N2, PD = 6; right: simulated DA609, PD = 12; n = 5000 agents each). **c** No PD navigation. **d** No roaming-dwelling switch. Playback speeds are accelerated by factors of 2000, 457, 389, and 24, respectively.

Supplementary Tables

Supplementary Table S1. Core model parameters

Source: M = matched to published *C. elegans* measurements; L = consistent with published ethogram or literature values; F = fitted to reproduce qualitative experimental dynamics.

Parameter	Value	Unit	Source	Model Role
Body length	1	mm	M[19]	Agent body dimensions
Segments per agent	20	—	M[19]	Aspect ratio 1:20; body geometry and collision
Segment spacing	0.05	mm	M[19]	1 mm ÷ 20 segments
Time step (Δt)	0.25	s	L[7]	Agent moves 1 segment per step at roaming speed
Steps per second (N2)	4	steps/s	L[7]	~4 body lengths/s roaming speed
Mean dwelling duration	25	s	F	More dwelling than roaming
Mean roaming duration	12.5	s	F	More dwelling than roaming
Dwelling steps (N2)	100	steps	F	25 s × 4 steps/s
Roaming steps (N2)	50	steps	F	12.5 s × 4 steps/s
PD — N2 (ρ_opt)	6	segments/pixel	F	Fitted to qualitative N2 wave morphology
PD — DA609 (ρ_opt)	12	segments/pixel	F	Fitted to qualitative higher PD than N2
Density penalty above optimal	3×	factor	F	Overcrowding penalized more than isolation
Angular noise (σ)	45	degrees	F	Directional persistence of locomotion
Food consumption rate (r)	0.999	per step	F	Calibrated to wave propagation timescale
Collective feeding efficiency	0.2	—	F	Sublinear scaling of multi-worm feeding
Odor decay radius	50	pixels	F	Approximate food gradient diffusion scale
Odor threshold	0.4	normalized	F	Minimum odor for chemotaxis activation
Arena size	30 × 30	mm	F	Matches experimental plate scale
Grid resolution	20	pixels/mm	F	Matches imaging resolution
Food patch size	21x12	mm	F	Matches experimental food lawn shape

Supplementary Table S2. Statistics. All group statistics and test results for quantitative comparisons reported in Figs. 2–4 and Supplementary Figs. S2 and S4. Permutation tests use 10,000 resamples. Entries in the Value columns represent Mean ± s.d. unless otherwise indicated (see individual rows for r, ρ, R^2, and fold-ratio entries).

Figure	Metric	Group 1	Mean ± SD	N	Group 2	Mean ± SD	N	Test	p
Fig. 2d	Nematic r, elongated	Before interaction	r = 0.11	868 pairs	After	r = 0.45	—	Rayleigh	< 0.001
Fig. 2d	Nematic r, round	Before interaction	r = 0.31	83 pairs	After	r = 0.31	—	Rayleigh	< 0.001
Fig. 3a	Wave speed (mm/h)	Exp N2	16.5 ± 3.0	14	Exp DA609	10.4 ± 3.9	8	Perm.	0.007
Fig. 3a	Wave speed (mm/h)	Sim N2	2.45 ± 0.025	20	Sim DA609	2.15 ± 0.50	20	Perm.	0.006
Fig. 3a	Wave bulkiness (mm)	Exp N2	2.13 ± 0.46	14	Exp DA609	1.67 ± 0.26	8	Perm.	0.022
Fig. 3a	Wave bulkiness (mm)	Sim N2	0.375 ± 0.016	20	Sim DA609	0.308 ± 0.016	20	Perm.	< 0.001
Fig. 3c	Large hole area (px^2)	Exp N2	4031 ± 3149	14	Exp DA609	2351 ± 974	8	Perm.	0.164
Fig. 3c	Large hole area (px^2)	Sim N2	192.9 ± 42.3	20	Sim DA609	473.1 ± 101.9	20	Perm.	< 0.001
Fig. 3c	TCI bulkiness (mm)	Exp N2	8.72 ± 4.15	14	Exp DA609	4.65 ± 1.38	8	Perm.	0.030
Fig. 3c	TCI bulkiness (mm)	Sim N2	0.780 ± 0.074	20	Sim DA609	0.734 ± 0.076	20	Perm.	0.066
Fig. 4a	Large hole area vs TCI bulkiness, within N2	—	ρ = −0.53	14	—	—	—	Spearman	< 0.001
Fig. 4a	Large hole area vs TCI bulkiness, within DA609	—	ρ = 0.05	8	—	—	—	Spearman	0.827
Fig. 4a	Large hole area SD ratio	N2	3.53×	14	DA609	—	8	Perm. (SD ratio)	0.032
Fig. 4a	TCI bulkiness SD ratio	N2	2.60×	14	DA609	—	8	Perm. (SD ratio)	0.023
Fig. 4b	TCI bulkiness vs N (sim)	—	ρ = 0.97	11	—	—	—	Spearman	< 0.001
Fig. 4b	Large hole area vs N (sim)	—	ρ = −0.53	11	—	—	—	Spearman	0.091

Supp. Fig. S2	Trial duration	N2	161.3 ± 55.5 min	14	DA609	192.7 ± 75.8 min	8	t-test	0.28
Supp. Fig. S2	Wave speed ~ duration	N2	R^2 = 0.18	14	—	—	—	Pearson	0.13
Supp. Fig. S2	Wave speed ~ duration	DA609	R^2 = 0.59	8	—	—	—	Pearson	0.026
Supp. Fig. S2	Wave bulkiness ~ duration	N2	R^2 = 0.02	14	—	—	—	Pearson	0.63
Supp. Fig. S2	Wave bulkiness ~ duration	DA609	R^2 = 0.26	7	—	—	—	Pearson	0.24
Supp. Fig. S2	Large hole area ~ duration	N2	R^2 = 0.024	14	—	—	—	Pearson	0.60
Supp. Fig. S2	Large hole area ~ duration	DA609	R^2 = 0.004	8	—	—	—	Pearson	0.88
Supp. Fig. S2	TCI bulkiness ~ duration	N2	R^2 = 0.019	14	—	—	—	Pearson	0.64
Supp. Fig. S2	TCI bulkiness ~ duration	DA609	R^2 = 0.021	8	—	—	—	Pearson	0.73
Supp. Fig. S4	Hole-rank stability, k = 1	—	ρ = 0.66	22	—	—	—	Spearman vs k = 5	—
Supp. Fig. S4	Hole-rank stability, k = 3	—	ρ = 0.87	22	—	—	—	Spearman vs k = 5	—
Supp. Fig. S4	Hole-rank stability, k = 5	—	ρ = 1.00	22	—	—	—	Spearman vs k = 5	—
Supp. Fig. S4	Hole-rank stability, k = 7	—	ρ = 0.97	22	—	—	—	Spearman vs k = 5	—
Supp. Fig. S4	Hole-rank stability, *k* = 10	—	ρ = 0.95	22	—	—	—	Spearman vs *k* = 5	—
Supp. Fig. S4	Hole-rank stability, *k* = 15	—	ρ = 0.91	22	—	—	—	Spearman vs *k* = 5	—

Supplementary Methods

1. Wave Property Extraction

Wave temporal boundaries. The mode first-visit frame index was computed within the leftmost 1% and rightmost 1% of the food-patch column range of the TCI, yielding automated wave start and end frames. For experiments with complex wave dynamics, these boundaries were refined by manual annotation.

Wave mask extraction. At 10-minute intervals from wave start to end, a wave mask was extracted from the corresponding raw video frame. Each frame was flat-field corrected, inverted, and binarized using Otsu's method restricted to pixels inside the food-patch boundary. Morphological cleaning used erosion and dilation with a disk structuring element (radius ~7 pixels at experimental imaging resolution, scaled proportionally for simulated data) to remove isolated noise and fill gaps. Residual components below a minimum area threshold were discarded.

Wave centerline. For each wave mask, the centerline was defined as the column-wise mean x-coordinate across all mask-positive rows, smoothed by a moving-average filter (window 200 pixels).

Wave speed. A single global speed was computed per experiment as the horizontal displacement between manually annotated start and end coordinates divided by the annotated time span (mm/h).

Wave bulkiness. The maximal inscribed radius of each frame's wave mask was computed as the maximum value of the Euclidean distance transform of the mask interior, converted to mm (30 px/mm for experimental data; 20 px/mm for simulation sets). Note that *wave bulkiness* is our primary lateral measure of wave morphology. Because the wave front advances non-uniformly, the wave boundary is locally irregular, making cross-sectional width sensitive to the specific sampling location and orientation along the centerline. *Wave bulkiness* (yellow circles in Figure 3b) captures wave extent in a way that is invariant to local shape irregularities, and is therefore a better measure than the standard *wave width* (the mean length of sampled normal cross-sections; green lines in Figure 3b) in our case.

2. Network Property Extraction

Flat-field correction. Each raw video frame was corrected for non-uniform illumination using a pre-computed flat-field estimate. Corrected frames were converted to double-precision floating point and normalized to [0,1].

Temporal median filtering and TCI generation. Corrected frames were sampled at 10-frame intervals (100 s at 0.1 fps). A sliding temporal median was computed over six consecutive sampled frames (~10-minute effective window). The TCI was generated by taking the pixel-wise minimum of these median-filtered values across the entire recording:

$$TCI(x,y) = \min_t \left[\operatorname{median}_{s \in window(t)} I(x,y,s) \right]$$

where I(x,y,s) is the corrected intensity at pixel (x,y) at sampled frames. This minimum-of-medians operation retains the darkest sustained signal at each location (corresponding to peak worm density), while the temporal median suppresses transient single-worm events. Parameter choice is validated empirically. See Supplementary Methods 2.1, TCI parameter validation.

Food patch masking. For experimental data, the food patch boundary was defined from manually annotated polygon coordinates. For simulation sets, the boundary was extracted directly from simulation geometry. All downstream analyses were restricted to pixels inside this mask.

Hole segmentation. Otsu's method was applied to TCI pixel values inside the food-patch mask to produce a binary image. Connected components in the inverted binary image (bright = worm-free

regions) were identified. Components were excluded if: (i) their area was below a minimum threshold (100 pixels at 30 px/mm for experimental data; scaled for simulated data to preserve structure at simulation resolution), or (ii) more than 15% of their perimeter coincided with the food-patch boundary.

Large hole area **(5th rank)**. Retained holes were sorted by area in descending order; the 5th-largest area was taken as the primary metric. Rank stability was validated across k = 1–15. Supplementary Methods 2.2, Hole-rank metric stability.

TCI bulkiness **(maximal inscribed radius)**. The maximum value of the Euclidean distance transform of the dark (worm-occupied) foreground mask in the TCI, converted to mm.

2.1 TCI parameter validation

The Temporal Composite Image (TCI) provides a cumulative spatial record of worm visitation by retaining, for each pixel, the minimum intensity observed across the entire experiment after temporal-median pre-filtering. Two design choices require justification: the use of minimum projection rather than an alternative summary statistic, and the application of a temporal median buffer before taking the minimum.

Minimum versus alternative projections. Time-averaged projections conflate transiently and persistently visited pixels: a location visited briefly once and one occupied throughout the experiment produce identical mean intensities, merging bundles (occupied areas) and holes (unoccupied areas) into a uniform grey field. Maximum-intensity projections highlight the brightest (worm-free) moments, inverting the biologically relevant signal. Minimum projection captures the darkest moment each pixel experienced, corresponding to its peak local worm density. Because worms are semi-transparent in our bright-field microscopy, this minimum directly encodes the most concentrated local occupancy, preserving channelized structure and enabling the distinction between persistently occupied bundles and under-visited holes that underlies all downstream structural metrics.

Temporal median buffering. Taking a pixel-wise minimum over all raw frames would cause isolated worms or brief imaging artifacts to register as persistent occupancy, artificially filling holes. A sliding temporal median filter—computed over 6 frames sampled every 10 raw frames at 0.1 fps (approximately one frame per 100 s), spanning a 10-minute window—suppresses these transient single-worm signals while retaining the sustained occupancy signature of the traveling wave. The minimum is then taken over the resulting median-filtered sequence, so only events persisting across the buffer window contribute to the TCI. The choice of parameters is justified empirically in Supplementary Figure S1.

Subsamples per 10-minute buffer. At a single subsample per buffer the median is computed from one frame and therefore fails to suppress transient individual-worm events: for the N2-high-*TCI bulkiness* case, *large hole area* is 4-fold overestimated (11.9 vs 3.1 mm² at default) and *TCI bulkiness* is 4-fold underestimated (3.3 vs 14.3 mm), because frames in which a single worm transiently darkens a hole are not averaged out and are therefore selected as the minimum, making holes appear spuriously small and bundles disconnected. From 6 subsamples onward, both metrics plateau for all three cases (maximum change between 6 and 60 subsamples: 18% for *large hole area*, 4% for *TCI bulkiness*). Increasing to 12 or 60 subsamples produces no meaningful additional change, confirming that 6 is not a boundary choice (Supplementary Figure S1a–b).

Buffer duration. At 1-minute buffers the window is too narrow to fully suppress transient worm visits: for the N2 (high *TCI bulkiness*) case, *large hole area* is 2.5-fold underestimated (1.25 vs 3.1 mm² at default), because brief individual worm appearances darken genuine hole pixels in the temporal median, and these artefacts survive the minimum projection. At 5 minutes, values approach but have not yet fully converged to the default. Above 10 minutes, over-aggregation sets in: as the buffer spans frames both before and after the wave has passed a location, the resulting median is brighter than the true wave-passage minimum, effectively erasing network contrast. Between 10 and 30 minutes, *large hole area* for the same case inflates 9-fold (3.1 vs. 28.3 mm²) as

brightened bundle pixels cross the Otsu threshold and are reclassified as holes, while *TCI bulkiness* collapses 6-fold (14.3 vs. 2.4 mm) as the worm-occupied region contracts correspondingly. The 10-minute default therefore sits between two failure modes: insufficient noise suppression at short durations and temporal over-aggregation at long durations (Supplementary Figure S1c–d).

Default parameters (6 subsamples, 10-minute buffer) are shown in Supplementary Figure S1e–g, with the 5th-largest hole (arrowhead) and *TCI bulkiness* (circle) indicated for each case. The single-subsample failure mode (Supplementary Figure S1h–j) and long-buffer failure mode (Supplementary Figure S1k–m) illustrate both extremes.

2.2 Hole-rank metric stability

Colour-coded rank matrices for all experiments (N2 and DA609 combined), ordered by the 5th-largest hole rank, show a consistent gradient pattern across columns $k = 3$ to $k = 15$, confirming that the relative ordering of experiments is preserved across this range (Supplementary Figure S3a). In contrast, the $k = 1$ column departs visibly from the gradient, indicating that the largest hole is an unreliable outlier — consistent with its susceptibility to transient imaging artifacts — and motivating the use of $k = 5$ as the representative *large hole area* metric.

Spearman rank correlation between the ordering produced by the 5th-largest hole and that produced by the kth-largest hole reaches 0.87 at $k = 3$, 1.0 at $k = 5$ by definition, and remains above 0.94 through $k = 7$, with a gradual decline to 0.91 at $k = 15$. The markedly lower value at $k = 1$ ($r = 0.66$) corroborates the rank-matrix pattern (Supplementary Figure S3b). Together, these analyses confirm that $k = 5$ sits within a stable regime ($k = 3$–10) and is not a cherry-picked value.

2.3 Independence of phenotypic metrics from trial duration

Trial durations ranged from 90 to 335 min. To rule out recording length as a confound, we computed the Pearson correlation between trial duration and each primary metric separately per strain (Supplementary Figure S2). Network metrics (*large hole area*, *TCI bulkiness*) showed no significant correlation with duration in either strain (all $R^2 < 0.03$, all $p > 0.59$). *Wave bulkiness* was uncorrelated with duration in N2 ($R^2 = 0.02$, $p = 0.63$) and non-significant in DA609 ($R^2 = 0.26$, $p = 0.24$; $n = 7$), where the elevated R^2 likely reflects the reduced sample size rather than a true trend. Manual *wave speed* was likewise uncorrelated with duration in N2 ($R^2 = 0.18$, $p = 0.13$), but showed a significant positive trend in DA609 ($R^2 = 0.59$, $p = 0.026$), consistent with kinetic maturation specific to that strain. Because trial durations were statistically matched between strains (N2: 161 ± 56 min; DA609: 193 ± 76 min; $p = 0.28$, two-sample t-test), this trend does not confound the reported inter-strain speed difference.

3. Agent-Based Simulation

The model implements the four components named in the main text as follows: (*i*) *body elongation*, the ring-buffer segment chain described under Agent body model; (*ii*) *roaming-dwelling switching*, the density-mismatch transition rules under Roaming-dwelling switch; (*iii*) *preferred density navigation*, the 8-neighbor density-gradient rule under Density-gradient navigation (movement priority 2); (*iv*) *food chemotaxis*, the odor-guided movement rule under Food chemotaxis (movement priority 1). The interaction logic of these components across the spatial arena is described in Section 4.

Spatial domain*.* The simulation arena is a 30 × 30 mm square on a 600 × 600 pixel grid (20 px/mm). Agent positions are stored as continuous floating-point coordinates; density calculations use floored integer pixel coordinates. The food patch, a rounded rectangle measuring 21x12 mm, occupies the vertically centered right portion of the arena to align with the experimental geometry. . The worm reservoir occupies an elliptical region on the left side, non-overlapping with the food patch.

Agent body model. Each agent is represented as a chain of 20 (x,y) coordinate pairs spaced ~50 µm apart, giving a total body length of ~1 mm and an aspect ratio of 1:20. At each step, a new head coordinate is added in the direction of movement and the rearmost (tail) segment is removed, so the chain advances while keeping body length constant. All 20 segments contribute to local density calculations. Throughout this section, PD (ρ_opt in equations) denotes the preferred local worm density parameter.

Round-agent variant. For elongation-necessity tests, agents arrange their 20 segments in a compact circular cluster centered on the head position, preserving total segment number while removing body anisotropy. A short history tail (~3 segments) is preserved to maintain a directional memory vector.

Roaming-dwelling switch. Each agent switches stochastically between dwelling and roaming. Per-step transition probabilities depend on a normalized density-mismatch score rel, computed as the sum of distFromOptimal values across all body-segment pixels, normalized by 20 × PD, where distFromOptimal = |local_density − PD| for under-dense pixels and 3 × (local_density − PD) for over-dense pixels. The transition probabilities are:

For $0 \le rel \le 1$:

$$p\_leave\_roam = (1 - rel) \times 1 + rel \times (1/avgRoamingSteps)$$

$$p\_leave\_dwell = (1 - rel) \times 0 + rel \times (1/avgDwellingSteps)$$

For $rel > 1$:

$$p\_leave\_roam = (1/avgRoamingSteps) \times \exp(-(rel - 1))$$

$$p\_leave\_dwell = (1/avgDwellingSteps) + (1 - 1/avgDwellingSteps) \times (1 - \exp(-(rel - 1)))$$

A minimum floor of 0.25 × the baseline rate is applied to both probabilities. Initial states are drawn from the stationary distribution.

Food chemotaxis (movement priority 1). An odor field is precomputed from the current food-patch state using a multi-threshold exponential-decay approximation. A roaming agent enters odor-guided mode with probability:

$$p\_guided = \sigma((foodThreshold - f) / softness) \times \sigma((s - odorThreshold) / softness)$$

where σ is the logistic function, f is local food concentration, s is local odor magnitude, *foodThreshold* = 0.2, *odorThreshold* = 0.4, *softness* = 0.05. An odor-guided agent moves one step in the local gradient direction plus angular noise drawn from uniform (−45°, +45°), clamped to arena bounds.

Density-gradient navigation (movement priority 2). Roaming agents not in odor-guided mode examine the 8 neighboring pixels. The neighbor with density closest to PD is the target. If a density gradient exists (neighbors are not all equal after removing self-overlapping segments), the agent steps toward that target; otherwise the agent continues in the current head-to-tail direction (priority 3: correlated random walk). Angular noise (σ = 45°) is added in all cases.

Food consumption. At each pixel with n worm heads present:

$$n\_eff = 1 + (n - 1) / (1 + (1/collectiveFeedingEfficiency) \times (n - 1))$$

Food concentration updates as $F \leftarrow F \times r^{n\_eff}$ with r = 0.999 and *collectiveFeedingEfficiency* = 0.2.

Simulation termination. Simulations were terminated when the residual food quantity fell below 50% of its initial value within the vertical stripe located 5% to 10% from the right edge of the arena. This criterion was selected to optimize the simulation duration relative to patch consumption while minimizing confounding edge effects.

Video output. Simulation state is rendered at 0.1 fps. Pixel intensity reflects local worm density, scaled by opacity = 0.1, inverted, and clipped to [0,1] to mimic bright-field microscopy.

Nematic alignment analysis. Pairwise interaction events were identified as two single-agent trajectories that briefly merged and then separated. Agent body-axis angles were measured immediately before and after separation. For elongated agents, distributions were corrected for geometric sampling bias by the cross-sectional width of two rectangular bodies at each approach angle. For round agents no geometric correction was applied, as circular bodies present an isotropic cross-section. In both cases a non-zero baseline r is expected from kinematic encounter bias: in a correlated random walk, head-on encounters occur at relative speed ≈ 2v and are over-represented, while same-direction encounters require one agent to be dwelling while the other overtakes (relative speed ≈ v) and are under-represented; both approach directions collapse to the same nematic angle, producing non-zero r independent of any alignment effect. The relevant quantity for assessing nematic alignment induced by interaction is therefore $\Delta r = r_{after} - r_{before}$ rather than absolute r. Mean resultant length *r*, a standard measure of directional concentration ranging from 0 (uniform) to 1 (perfect alignment), was computed from the corrected distribution.

4. Numerical illustration of component interactions

The three behavioural components — roaming-dwelling switch, PD navigation, and food chemotaxis — do not act independently; their interaction produces distinct effective behaviours across the arena that shift dynamically as food is consumed. Three characteristic situations illustrate this.

Worm inside a cluster at PD, away from food. When local density matches PD (normalized mismatch *rel* = 0), the per-step probability of leaving the dwelling state collapses to its minimum floor (~0.003, mean dwelling about ~100 s for N2 parameters) while the probability of leaving roaming approaches 1. A roaming worm arriving at PD therefore dwells within one or two steps (~0.5 s). Among the small roaming fraction permitted by the floor, chemotaxis activates with probability proportional to odor magnitude: the odor field decays exponentially with distance *d* from the food boundary ($\beta \approx 0.17\ mm^{-1}$), reaching the 50% activation threshold at $d^* \approx 5.4$ mm and falling below 10% beyond ~7 mm. At the reservoir center (~5–6 mm from food), a worm in a cluster therefore experiences: p(dwell this step) ≈ 0.997, p(chemotax | roaming) ≈ 0.5, yielding an effective per-step probability of moving toward food of roughly 0.003 × 0.5 ≈ 0.15%. The cluster is essentially static.

Worm in the wave front on intact food. A cluster arriving at a food-rich location remains near PD (dwells for the same reason as above). Chemotaxis is additionally suppressed by a separate food-availability factor pFood, implemented as a logistic function that approaches zero when local food concentration exceeds ~0.2 (normalized): at fully intact food levels (~1.0), *pFood* ≈ 0.018, reducing p_guided by ~50-fold regardless of odor strength. Worms on intact food therefore have an effective chemotaxis probability of ~0.003 × 0.018 × *p_odor* ≈ 0.005% — effectively zero. They dwell and eat.

Worm in the wave front as food depletes. Food consumption reduces local food concentration progressively at each step. As the local value falls below 0.2, *pFood* rises above 0.5 and approaches 1 at full depletion. Simultaneously, the odor field — recomputed each step from the current food distribution — now carries a gradient pointing toward intact food ahead. The floor-rate roaming trickle remains the only eligible pool (~0.3% of steps), but among those roaming steps, chemotaxis guidance now rises steeply: at 50% local depletion and 5 mm from the food edge, *p_guided* ≈ 0.003 × 0.5 × 0.5 ≈ 0.075% per step, and at full depletion *p_guided* ≈ 0.003 × 0.98 × 0.5 ≈ 0.15%. This gentle but consistent bias draws the small roaming fraction toward the receding

food edge, pulling the cluster forward gradually as food disappears beneath it. The wave therefore propagates not by worms breaking away from the cluster, but by the cluster being gently steered forward at floor-rate by the combined release of chemotaxis suppression and the shifting odor gradient.

The behavioural regimes across the arena with cluster stasis, food-pinned dwelling, and depletion-gated forward pull, are therefore not pre-specified spatial zones but emerge dynamically from the interaction of the three components with the evolving food distribution.

5. Mechanism tests

Figure 2d–g shows four matched simulations in which a single model component is removed while all other parameters remain as listed in Supplementary Table S1 (1500 agents, default rectangular arena and food geometry). Supplementary Video S3 shows the full dynamics of all four conditions side by side.

Body elongation (Figure 2d; Supplementary Video S3a). Round-body agents retain the same 20 body segments and all identical parameters as elongated agents. The sole change is how the segments are spatially arranged: instead of a chain forming a 1:20 aspect-ratio body, segments are distributed in a compact circular cluster around the head, occupying the same total area. This removes the geometric anisotropy responsible for nematic alignment without altering agent number, density sensing, roaming-dwelling switch, or chemotaxis. Angular distribution analysis confirmed that pairwise interactions increased alignment exclusively for elongated agents. For these agents, the mean resultant length increased from $r = 0.11$ before interactions to $r = 0.45$ after ($\Delta r = 0.34$). In contrast, round agents showed negligible change ($\Delta r = 0.007$), consistent with an absence of interaction-induced alignment. The non-zero baseline ($r = 0.31$) for round agents reflects a kinematic encounter bias from correlated locomotion rather than true nematic alignment, as head-on encounters were over-represented relative to same-direction meetings. Without elongation, agents consume the food patch from all directions in a uniform advancing front with no channelized wave or network imprint.

Food chemotaxis (Figure 2e; Supplementary Video S3b). The food chemotaxis component is disabled: the food gradient is computed but agents do not navigate toward it. Agents form spontaneously evolving dynamic networks but no traveling wave, confirming that directed chemotaxis, not merely food presence, is required for wave formation. Notably, when chemotaxis is removed but PD is varied between strains (Supplementary Video S3b shows N2 PD = 6 left, DA609 PD = 12 right), network topology still differs: the higher PD of the simulated DA609 produces denser, more interconnected aggregates despite identical food environments. This confirms that network topology is governed by PD independently of food-driven dynamics.

Density preference (Figure 2f; Supplementary Video S3c). Density-modulated movement is disabled so all locations are treated as equally attractive regardless of local worm density. Agents navigate by chemotaxis alone. The resulting TCI shows agents distributed approximately uniformly across the food patch, with a slight intensity increase near the patch boundaries, and no network imprint, confirming that density-dependent aggregation is necessary for bundle and hole formation.

Dwelling state (Figure 2g; Supplementary Video S3d). The dwelling state is disabled so agents transition immediately back to roaming upon stopping. Roaming-only agents initially produce a wave front and a transient network imprint, but these collective structures subsequently fragment into randomly distributed short-lived aggregates. The dwelling state, and the local density amplification it provides, is therefore necessary for sustained network maintenance.

Supplementary Notes

Supplementary Note S1. Biological realism of model parameters and acknowledged quantitative limitations

The simulation was designed to identify the components sufficient to generate the observed collective dynamics and relative between-strain differences, rather than to reproduce absolute metric values.

Parameter grounding. Body dimensions (1 mm total length across 20 segments, ~50 μm width per segment) match published adult *C. elegans* measurements[21]. The integration time step (Δt = 0.25 s) satisfies the constraint that an agent moving at typical roaming speed (~4 body lengths s^{-1}) displaces less than one segment per step. While simplified for simulation step-size consistency, our assigned durations for dwelling and roaming (25 s and 12.5 s) qualitatively align with the behavioural partitioning described in published ethograms on bacterial lawns, where the dwelling state is the predominant phase[20]. The strain-specific PD parameter (PD = 6 segments $pixel^{-1}$ for N2; PD = 12 for DA609) reflects the enhanced aggregation phenotype conferred by the *npr-1* null mutation in DA609; its value was determined by matching observed wave morphology rather than by independent measurement. All parameters are listed in Supplementary Table S1.

Scale of quantitative discrepancy. Absolute metric values in the simulations differ from experiments by approximately one order of magnitude across metrics (Supplementary Table S2): *wave speed* ~7× lower (experimental N2: 16.5 ± 3.0 vs simulated N2: 2.5 ± 0.03); *wave bulkiness* ~6× lower (2.1 ± 0.46 vs 0.38 ± 0.016 mm); *large hole area* ~21× lower (4031 ± 3149 vs 193 ± 42 px^2); *TCI bulkiness* ~11× lower (8.7 ± 4.2 vs 0.78 ± 0.074 mm).

Interpretation. We prioritized identifying the components sufficient to generate the observed behavioural repertoire rather than fitting model parameters to collective-level outputs. Because none of the emergent metrics — *wave speed*, *wave bulkiness*, *TCI bulkiness*, *large hole area*, strain differences, or N2 variability structure — was used to constrain the parameters, their concordance across four independent comparisons reflects the explanatory reach of the four identified model components rather than parameter absorption. Systematic calibration to absolute values would be a valuable next step: it could identify which specific aspects (e.g. unreported density-sensing thresholds, biophysical constraints on body mechanics, or food-patch geometry differences) account for the quantitative gap, and in doing so would deepen mechanistic understanding beyond what component-level identification alone achieves. Whether the quantitative gap between experiments and simulations reflects a missing mechanism or an unexplored parameter region remains an open question. The simulation reproduces: (i) the structural network topology and full wave phenomenology; (ii) the direction and statistical significance of strain differences in all primary wave metrics; and (iii) the characteristic per-trial variability of N2 in contrast to the more stereotyped DA609 cryptic network topology. This concordance across multiple independent features supports the conclusion that density-dependent roaming-dwelling switch and elongation-induced nematic alignment are the dominant drivers of our collective behaviour.

Supplementary Note S2: Open questions and directions for future investigation

Three sets of questions follow from the findings that the current study opens but does not resolve.

Network-to-wave transition mechanism. The transition between wurmuration phases — reservoir drainage, wave initiation, and eventual collective dissolution — is characterized here at the macroscale through TCI and wave metrics. The individual-level dynamics of this transition remain uncharacterized: whether network dissolution is triggered by a threshold in local food odor, a critical reduction in reservoir density, or a temporal accumulation of individual chemotaxis-driven departures is not resolved by the current data. Multi-worm tracking at population scale across both phases could address these questions and determine whether the transition has the character of a sharp collective switch or a gradual crossover.

Sources of N2 per-trial variability. The scatter in N2 network topology metrics is consistent with per-trial variation in the fraction of the population actively participating in collective movement. This predicts that per-trial variation in the fraction of the population entering the food patch should correlate with TCI topology — a prediction testable with existing bright-field imaging methods using the partial optical opacity of *C. elegans*.

Model calibration. The simulations reproduce directional trends and relative strain differences across all primary metrics but differ from absolute experimental values by approximately one order of magnitude (Supplementary Note S1). Closing this gap requires independent measurement of the biological quantities the model treats as free parameters: the density deviation thresholds governing roaming-dwelling transition probabilities, the spatial range of food odor sensing, and the effective density footprint of individual body geometry. An allelic series titrating *npr-1* activity would map the molecular-to-behavioural axis of PD and enable calibration from measured phenotype rather than qualitative fitting to collective outcomes, which would also clarify whether the quantitative gap reflects missing mechanisms or unexplored parameter regimes.
